# The role of cell-matrix adhesion and cell migration in breast tumor growth and progression

**DOI:** 10.3389/fcell.2024.1339251

**Published:** 2024-02-05

**Authors:** Lor Huai Chong, Ai Kia Yip, Hui Jia Farm, Lamees N. Mahmoud, Yukai Zeng, Keng-Hwee Chiam

**Affiliations:** ^1^ Bioinformatics Institute, ASTAR, Singapore, Singapore; ^2^ School of Pharmacy, Monash University Malaysia, Bandar Sunway, Selangor, Malaysia; ^3^ Department of Computer Science, University of Oxford, Oxford, United Kingdom; ^4^ Biomedical Engineering Department, Faculty of Engineering, Helwan University, Helwan, Cairo, Egypt

**Keywords:** actomyosin contractility, 3D matrix rigidity, MCF7 spheroids, collagen matrix, agarose matrix, spheroids growth

## Abstract

During breast cancer progression, there is typically increased collagen deposition resulting in elevated extracellular matrix rigidity. This results in changes to cell-matrix adhesion and cell migration, impacting processes such as the epithelial-mesenchymal transition (EMT) and metastasis. We aim to investigate the roles of cell-matrix adhesion and cell migration on breast tumor growth and progression by studying the impacts of different types of extracellular matrices and their rigidities. We embedded MCF7 spheroids within three-dimensional (3D) collagen matrices and agarose matrices. MCF7 cells adhere to collagen but not agarose. Contrasting the results between these two matrices allows us to infer the role of cell-matrix adhesion. We found that MCF7 spheroids exhibited the fastest growth rate when embedded in a collagen matrix with a rigidity of 5.1 kPa (0.5 mg/mL collagen), whereas, for the agarose matrix, the rigidity for the fastest growth rate is 15 kPa (1.0% agarose) instead. This discrepancy is attributable to the presence of cell adhesion molecules in the collagen matrix, which initiates collagen matrix remodeling and facilitates cell migration from the tumor through the EMT. As breast tumors do not adhere to agarose matrices, it is suitable to simulate the cell-cell interactions during the early stage of breast tumor growth. We conducted further analysis to characterize the stresses exerted by the expanding spheroid on the agarose matrix. We identified two distinct MCF7 cell populations, namely, those that are non-dividing and those that are dividing, which exerted low and high expansion stresses on the agarose matrix, respectively. We confirmed this using Western blot which showed the upregulation of proliferating cell nuclear antigen, a proliferation marker, in spheroids grown in the 1.0% agarose (≈13 kPa). By treating the embedded MCF7 spheroids with an inhibitor or activator of myosin contractility, we showed that the optimum spheroids’ growth can be increased or decreased, respectively. This finding suggests that tumor growth in the early stage, where cell-cell interaction is more prominent, is determined by actomyosin tension, which alters cell rounding pressure during cell division. However, when breast tumors begin generating collagen into the surrounding matrix, collagen remodeling triggers EMT to promote cell migration and invasion, ultimately leading to metastasis.

## Introduction

Biological cells are exposed to a variety of mechanical factors, for example, matrix rigidity, porosity, and topography, in the extracellular matrix (ECM) (Butcher et al., 2009). Prior studies have shown that these mechanical factors can influence cell migration and adhesion behavior (Friedl, 2004; Zaman et al., 2006; Yip et al., 2015), cell growth, (Tilghman et al., 2010; Yeh et al., 2012), cell apoptosis (Cheng et al., 2009; Leight et al., 2012), and stem cell differentiation (Engler et al., 2006; Yim et al., 2007; Ankam et al., 2013). However, most of these studies have been conducted on either single cells or on two-dimensional (2D) substrates, which may not fully represent the three-dimensional (3D) environment of tissues in the body exist in. The response of cell clusters or multicellular spheroids to their external 3D mechanical environment has been less understood.

The understanding of multicellular response to mechanical factors in the 3D ECM is important in the context of tumor mass growth during cancer progression. The ECM is a critical component of the tumor microenvironment, consisting of fibrillar or non-fibrillar collagens, laminins, fibronectins, and other matrix proteins that support tumor development and progression (Mouw et al., 2014). For example, during tumor mass growth, cancer cells have to proliferate and expand against the mechanical stress imposed by the ECM (Montel et al., 2012). It has been shown that during breast cancer progression, mammographically dense breast tissue, due to increased fibrillar collagen deposition, is correlated strongly with a higher risk of developing breast carcinoma (Provenzano et al., 2008). The increased collagen-matrix density resulted in a high matrix concentration, and increased cell-matrix adhesion has been found to promote cancer cell growth and migration (Provenzano et al., 2009). Despite the possible link between ECM rigidity and cell growth, there have been limited studies examining how ECM concentrations and rigidity affect cell-matrix adhesion and migration during 3D breast tumor mass growth and progression.

To recapitulate the *in vivo* solid tumor architecture and biology, tumor cells can be grown as 3D multicellular spheroids *in vitro* using ultra-low attachment plates (Sant and Johnston, 2017). It has been shown that the 3D spheroids can reproduce some features of the tumor *in vivo*, for example, cell-cell interactions and Epithelial to Mesenchymal Transition (EMT) (Kimlin et al., 2013; Zanoni et al., 2016). However, spheroids cultured unconstrained in suspension in the culture media do not experience the compressive stress exerted by the ECM on the expanding spheroid. By adding a biocompatible polymer, Dextran, to the culture media to impose mechanical compressive stress on the spheroids, Delarue et al (2014) *.* and Montel et al. (2012) have shown that the spheroids growth can be reduced by mechanical compression, due to an increase in the cell proliferation inhibitor p27^Kip1^ (Montel et al., 2012; Delarue et al., 2014).

Spheroids can also be embedded in a 3D matrix, such as collagen gel or agarose gel, to study the effect of mechanical confinement and compression on tumor spheroid growth (Gordon et al., 2003; Plodinec et al., 2012; Desmaison et al., 2018). Desmaison et al. (2018) have shown that physically confining tumor spheroids in agarose gel altered cell nuclei geometry and induced prometaphase delay as compared to freely growing spheroids (Desmaison et al., 2018). In another study, Charoen et al. (2014) found that the growth rate of the tumor spheroids can be altered by changing the collagen concentration (2–5 mg/mL) of the gel, thereby changing the matrix’s mechanical properties, *e.g.*, matrix rigidity and porosity (Plodinec et al., 2012). Specifically, the authors found that bone cancer spheroids showed the highest cell growth in an intermediate collagen concentration (3–4 mg/mL), while breast cancer spheroids showed the fastest growth in the lowest collagen concentration (2 mg/mL). These results suggest matrix concentration and rigidity could play a role in modulating tumor spheroid growth.

In this study, we have investigated the effects of matrix concentration on the cell growth of breast tumor spheroids by embedding spheroids of MCF7, a breast adenocarcinoma cancer cell line, in both collagen and agarose gels of various concentrations which contribute to different rigidities. We first employ collagen matrices to mimic the tumor microenvironment of breast cancer progression as collagen deposition increases. We found that the MCF7 spheroids had the fastest cell growth at the lowest collagen matrix concentration (0.5 mg/mL ≈ 5.1 kPa). This observation is not only dependent on increased cell growth and reduced apoptosis events in the low collagen matrix concentration, but also an increase in EMT. This is because cells from the tumor spheroid can adhere to the collagen gel, creating cell-matrix adhesion, which allows them to migrate away from the spheroid cluster through EMT. While the collagen gel is physiologically relevant to the tumor microenvironment, cells can also remodel the collagen matrix by secreting matrix metalloproteinases (MMP) to degrade the collagen matrix, leading to multifaceted changes in matrix mechanical properties including matrix rigidity, matrix pore size, and cell-matrix adhesiveness (Bonnans et al., 2014). Therefore, it is challenging to attribute the changes in spheroid growth to matrix concentration and rigidity alone.

To address these problems, we embedded MCF7 spheroids within agarose gels because cells do not adhere directly to agarose, thereby impeding their cell-matrix adhesion and hindering their migration away from the tumor spheroid through EMT (Sant and Johnston, 2017). Therefore, it not only enables us to recapitulate cell-cell interactions that occur in the early stage of breast tumor growth, but also offers us a more profound insight into how alterations in matrix concentration and rigidity influence spheroid growth. Besides, agarose is an elastic matrix that cannot be remodeled or degraded by MMP, hence making it a suitable material to measure expansion stresses exerted by the growing spheroids on the ECM (Ahearne et al., 2005; Bonnans et al., 2014).

Interestingly, we have found that the MCF7 spheroid growth is the fastest at an intermediate agarose concentration (1.0% ≈ 13 kPa). By characterizing stresses exerted from the expanding spheroid on the agarose gel, we found that stresses exerted by the cells on the spheroid surface can be classified into two distinct populations and speculated that the two populations arise from non-dividing and dividing cells, which exert low and high expansion stress on the agarose gel respectively. In accordance with the optimum cell growth at intermediate agarose rigidity, we found a higher proportion of cells exerting high expansion stress in the spheroids embedded in intermediate agarose concentration (1.0% ≈ 15 kPa) as compared to the low (0.6% ≈ 8 kPa) or high (1.5% ≈ 20 kPa) agarose concentration and rigidity. This result suggested that the presence of more dividing cells in these spheroids in intermediate agarose concentration. This hypothesis was then verified through Western blots showing the upregulation of the proliferation marker proliferating cell nuclear antigen (PCNA) in spheroids grown in the intermediate agarose gel concentration and rigidity. We hypothesized that the optimum ECM concentration and rigidity observed for the fastest spheroid growth is related to cell cortex rigidity that determines the cell rounding pressure during cell division. Through the use of an inhibitor or activator of myosin contractility, we showed that the optimum rigidity for the fastest spheroid growth can be increased or decreased respectively.

## Results

### Spheroids are smaller and more circular in response to the increasing collagen concentrations

It has been shown that during breast cancer progression, collagen deposition increases, resulting in a higher matrix concentration and rigidity as well as increased cell-matrix adhesion. These factors have been shown to affect the cancer cell growth and EMT (Provenzano et al., 2008; Provenzano et al., 2009). To recapitulate the *in vivo* environment of the breast tumor mass, MCF7 human breast adenocarcinoma multicellular spheroids were embedded in 3D collagen matrix of varying collagen concentration (0.5–2.0 mg/mL), corresponding to matrix rigidity of 5–18 kPa (Roeder et al., 2002; Provenzano et al., 2009).

**FIGURE 1:**
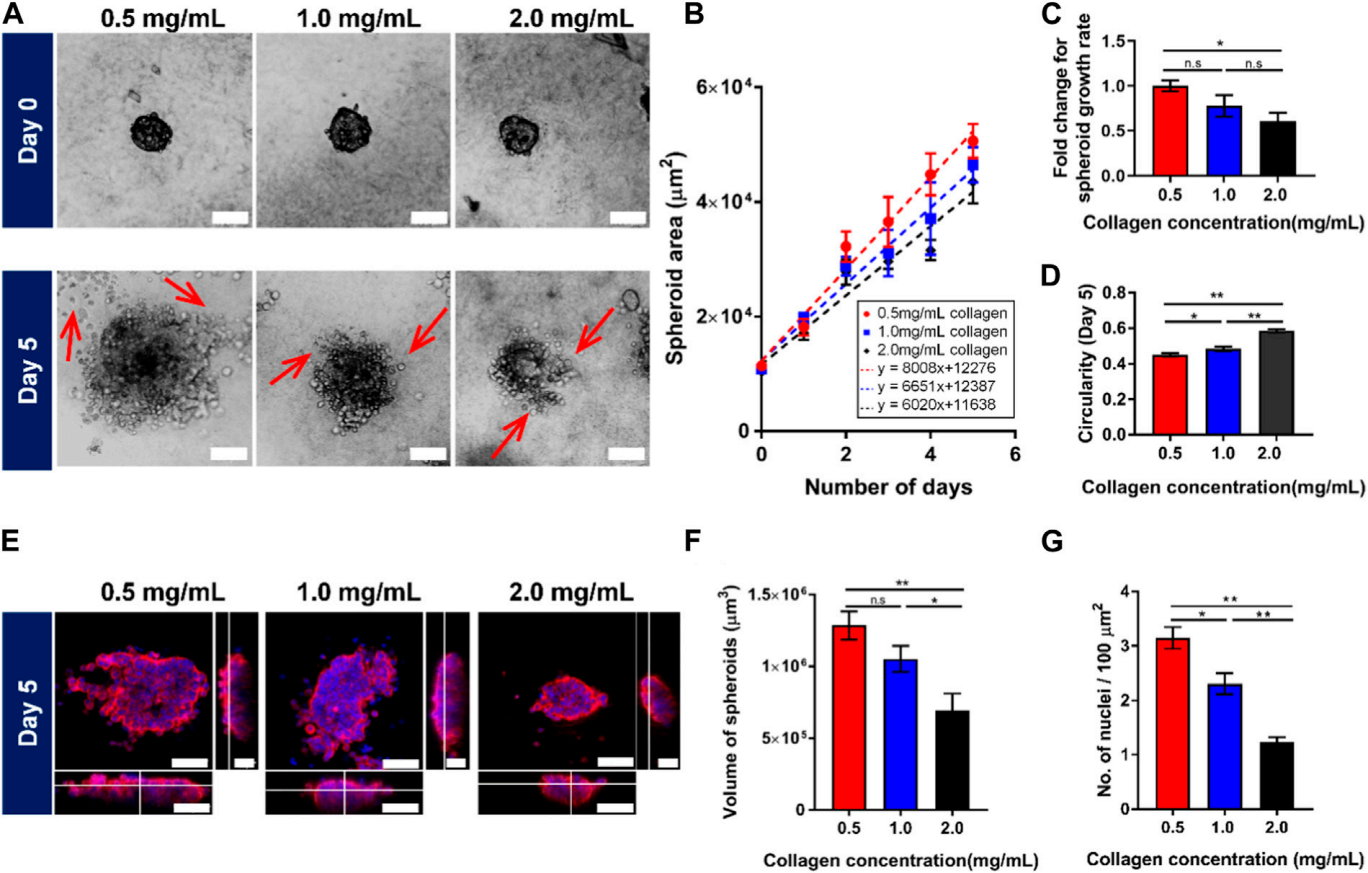
Spheroids embedded in 3D collagen matrix show lower growth rate with more circular morphology in response to the increasing collagen concentrations. **(A)** Brightfield images of spheroids embedded in 3D collagen matrices in different concentrations on Day 0 (top panel) and Day 5 (bottom panel). Red arrows denote the spreading of cells from MCF7 tumor spheroids. **(B)** Quantification of the spheroid area against the number of days the spheroids are embedded within the 3D collagen gel (n = 112, 102, 97 for 0.5 mg/mL, 1.0 mg/mL, and 2.0 mg/mL collagen concentrations respectively). **(C)** Fold change in spheroid growth rate for different collagen matrices concentration, normalized to the value for 0.5 mg/mL collagen concentration. **(D)** Quantification of the spheroids’ circularity embedded in different collagen concentrations on Day 5 (n = 112, 102, 97 for 0.5 mg/mL, 1.0 mg/mL, and 2.0 mg/mL collagen concentrations respectively). **(E)** Confocal images of 3D spheroids embedded in different collagen concentrations of 3D collagen matrix on Day 5, stained with the 4′,6-diamidino-2-phenylindole (DAPI) (blue), and F-actin (red). **(F)** Quantification of the volume of spheroids embedded in different collagen concentrations (n = 30, 37, 40 for 0.5 mg/mL, 1.0 mg/mL, and 2.0 mg/mL collagen concentrations respectively). **(G)** Quantification of nuclei obtained from 3D spheroids (n = 30, 37, 40 for 0.5 mg/mL, 1.0 mg/mL, and 2.0 mg/mL collagen concentrations respectively). The red bars denote 3D spheroids culture in 0.5 mg/mL of collagen gel, blue bars show 3D spheroids culture in 1.0 mg/mL collagen gel while black bars denote 3D spheroids culture in 2.0 mg/mL collagen gel. Data are average ±SD of 3 independent experiments. Asterisks denote the statistically significant differences (student t-test, *p < 0.05; **p < 0.001, n.s = no significant differences). The scale bar represents 100 μm.

The spheroids were imaged over a 5-day period on a phase contrast microscope (Figure 1A) and segmented to obtain the projected spheroid area. The spheroid areas were found to increase over time for spheroids in all three collagen matrix concentrations, 0.5 mg/mL, 1.0 mg/mL, 2.0 mg/mL (Figure 1B). The quantification of spheroid area against time fitted well to a linear relationship and the gradients of the linear fits were measured as the spheroid growth. We observed that the spheroid growth at the fastest rate (growth rate = 8,008 μm^2^/day) at the lowest collagen matrix concentration of 0.5 mg/mL (≈5.1 kPa) as compared to the other collagen matrix concentrations (growth rate = 6,651 μm^2^/day and 6,020 μm^2^/day) for collagen matrix concentration of 1.0 mg/mL (≈11.4 kPa) and 2.0 mg/mL (≈17.9 kPa), respectively (Figure 1C).

In high collagen matrix concentration (2.0 mg/mL ≈ 17.9 kPa), MCF7 spheroids also exhibited a more circular morphology (circularity index: 0.59 ± 0.02), compared to 0.48 ± 0.02 and 0.43 ± 0.02 at the intermediate and low collagen matrix concentration (Figure 1D). Consistent with spheroid area decrease, confocal z-stack images of MCF7 spheroids in collagen gel showed a significant reduction in spheroid volume with increasing collagen matrix concentration (about 50% decrease on average for the highest collagen matrix concentration (2.0 mg/mL) *versus* the lowest collagen matrix concentration (0.5 mg/mL)) in Figures 1E, F. We also observed that fewer cells were found in the spheroids grown in a high collagen matrix concentration (1.22 ± 0.11 nuclei/100 μm^2^) compared to the lowest collagen matrix concentration, which was about 3.14 ± 0.2 nuclei/100 μm^2^ (Figure 1G). Taken all these together, our results demonstrated that MCF7 spheroids showed attenuated growth and more circular morphology with increasing collagen matrix concentrations.

### Growing tumor spheroid demonstrates decreasing trends in cell growth and EMT as well as increasing trends in cell cycle arrest and apoptosis in response to the increasing collagen concentration

**FIGURE 2:**
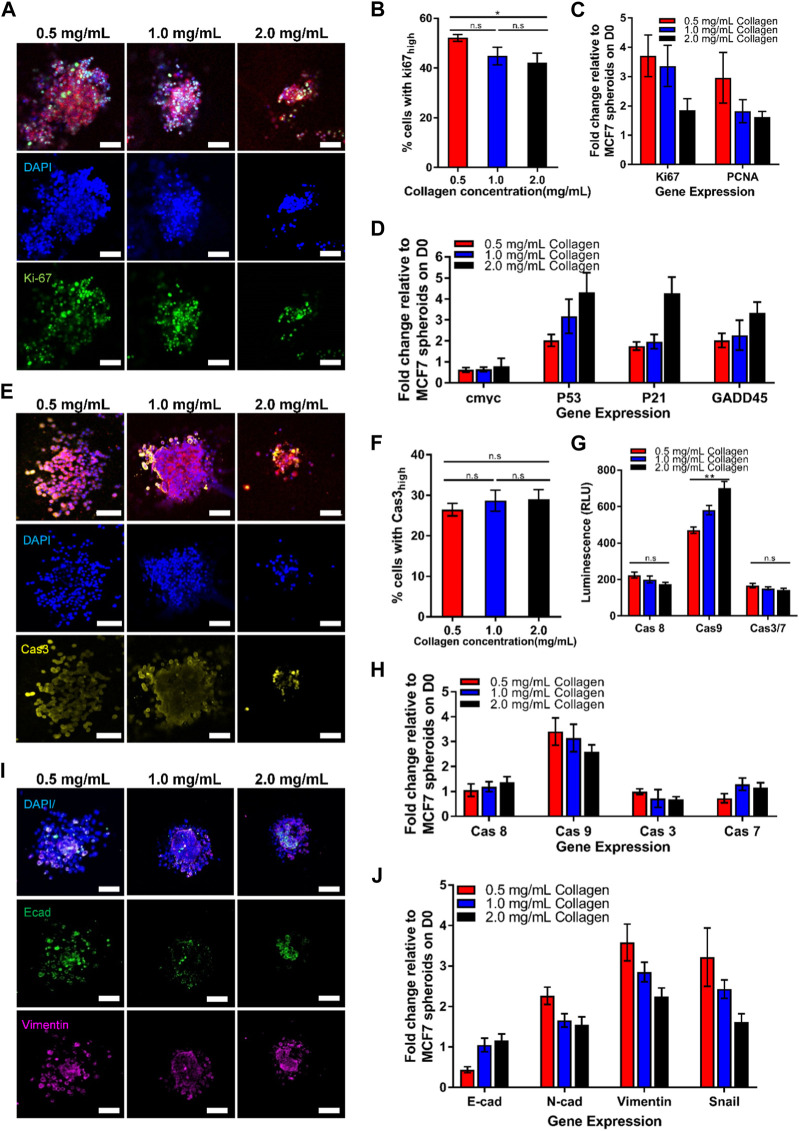
Growing tumor spheroid in the increasing collagen matrix lowers cell growth, increases cell cycle arrest and apoptosis, as well as decreases the epithelial-mesenchymal transition (EMT) process. Representative confocal images of MCF7 spheroids grown in different collagen concentrations for **(A)** cell growth markers, **(E)** apoptosis markers, and **(I)** EMT markers on Day 5. **(A)** Confocal images of 3D spheroids embedded in different collagen concentrations, stained with nuclei (blue), F-actin (red), and Ki67 (green). **(B)** Quantification of nuclear staining intensity of Ki67 (n = 24, 27, 30 for 0.5 mg/mL, 1.0 mg/mL, and 2.0 mg/mL collagen concentrations respectively). **(C)** Gene expression of cell growth markers. **(D)** Gene expression of cell cycle arrest markers. **(E)** Confocal images of 3D spheroids embedded in different collagen concentrations, stained with nuclei (blue), F-actin (red), and Caspase 3 (yellow). **(F)** Quantification of nuclear staining intensity of Caspase 3 (n = 19, 23, 27 for 0.5 mg/mL, 1.0 mg/mL, and 2.0 mg/mL collagen concentrations respectively). **(G)** Luminescence level of caspases activities. **(H)** Gene expression for apoptosis. **(I)** Confocal images of 3D spheroids embedded in different collagen concentrations, stained with nuclei (blue), E-cadherin (green), and Vimentin (pink). The red bars denote 3D spheroids culture in 0.5 mg/mL of collagen gel, blue bars show 3D spheroids culture in 1.0 mg/mL collagen gel while black bars denote 3D spheroids culture in 2.0 mg/mL collagen gel. Data are average ±SD. Asterisks denote the statistically significant differences (student t-test, *p < 0.05; **p < 0.001, n.s = no significant differences). Scale bars represent 100 μm. **(J)** Gene expression of EMT markers.

To investigate the possible mechanisms causing the attenuated spheroid growth in response to the increasing collagen matrix concentrations, we sought to examine the potential of collagen matrix concentrations to regulate cell growth, cell cycle arrest, apoptosis and EMT. Firstly, Ki67 and PCNA were selected as markers for cell growth in spheroids tumor, which are associated with the cell cycle, especially during the late G1, S, G2, and M phases, when the cells are rapidly growing and dividing. Quantitative analysis of the nuclear staining of Ki67 in Figures 2A, B revealed a decreased percentage of proliferating cells with Ki67 markers (42.13% ± 3.83%) in high collagen matrix concentration (2.0 mg/mL ≈ 17.9 kPa) compared to 52.14% ± 1.4% in low collagen matrix concentration (0.5 mg/mL ≈ 5.1 kPa). Similar results were obtained for the transcriptional levels of Ki67 and PCNA, at which, both genes demonstrated significant decreasing trends with the increasing collagen matrix concentrations from 0.5 mg/mL (≈5.1 kPa) to 2.0 mg/mL (≈17.9 kPa) (Figure 2C). This data suggested that the high concentration of collagen matrix reduced the growth of the tumor spheroids, resulting in smaller size of MCF7 spheroids.

Next, we aimed to study if the collagen matrix concentration could affect the cell cycle and apoptosis of MCF7 tumor spheroids. The p53 gene has been previously described to regulate the cell cycle (Chen, 2016; Aubrey et al., 2018). Therefore, we examined the transcriptional level of p53 and its downstream targets, including p21 and GADD54, each of which independently exhibit proliferation-suppressive activity. P21 inhibits cell cycle progression into the S phase through Cdk2, and/or PCNA (Funk et al., 1997; Cayrol et al., 1998; Abbas and Dutta, 2009), while GADD54 inhibits the progression into the M phase through Cdc2 (Vairapandi et al., 2002; Casimiro et al., 2012). Our data in Figure 2D revealed that p53 was elevated two folds with the increasing concentration of collagen matrix. Similarly, the downstream p53 targets, including p21 and GADD54, demonstrated relatively higher expression levels corresponding to the increased p53 transcriptional levels in high collagen matrix concentration (2.0 mg/mL ≈ 17.9 kPa), showing similar trends with p53 transcriptional level. Overall, these results suggested that increased collagen matrix concentration attenuated cell growth by triggering p53 levels which inhibited the cell cycle progression through p21 and GADD54, contributing to smaller spheroids sizes in high collagen matrix concentration.

In our study, we prioritize apoptosis over necrosis as the primary mode of cell death. This choice is driven by the observation that necrosis tends to manifest in larger solid tumors exceeding 4 mm in diameter, characterized by the presence of necrotic cells in the core regions of the tumor (Li et al., 2007; Lee et al., 2018). As such, it is not directly relevant to our study because our spheroids are generally less than 1 mm in diameter. In addition, necrosis typically results from extreme conditions, such as exposure to toxins, elevated temperatures, and reduced oxygen levels (Liu and Jiao, 2019), which are not the main focus of our study. Therefore, our primary emphasis lies in evaluating apoptosis as the mechanism of cell death because apoptosis is closely associated with the tumor growth and progression (Su et al., 2015).

For apoptosis, we examined both the initiator caspases (caspase 8 and 9) and executioner caspases (caspase 3 and 7) activities. The confocal images in Figure 2E demonstrated that there was no significant difference in activated caspase 3 expression with the increasing of collagen matrix concentration (Figures 2E, F). We then used Promega Caspase-Glo kits and RT-PCR to detect the activation of the caspase’s activities, including caspases 8, 9, 3, 7. Interestingly, we found that only caspase 9 activity showed significant differences with increasing collagen matrix concentration (Figure 2G) but not in caspase 8, 3, and 7. The high collagen matrix concentration (2.0 mg/mL ≈ 17.9 kPa) demonstrated the highest caspase 9 levels, which was 469.75 ± 17.82 (RLU), followed by 579.83 ± 25.20 (RLU) in the intermediate collagen matrix concentration (1.0 mg/mL ≈ 11.4 kPa), and 700.5 ± 36.6 (RLU) in the low collagen matrix concentration (0.5 mg/mL ≈ 5.1 kPa). This result was verified through the gene expression data in Figure 2H, showing the highest expression of the caspase 9 gene expression, which was 3.4 ± 0.55 folds in high collagen matrix concentration (2.0 mg/mL ≈ 17.9 kPa) compared to 2.6 ± 0.27 folds in low collagen matrix concentration (0.5 mg/mL ≈ 5.1 kPa). We further found that the caspase 9 demonstrated a similar trend with the response of p53 towards the collagen matrix concentration, showing that the caspase 9 activation may be due to the regulation of p53. Previous studies have proved that p53 is a sensor of cellular stress and is a critical activator in regulating caspase 9 to initiate intrinsic apoptosis pathway (Soengas et al., 1999; Wu and Ding, 2002). Therefore, our finding suggested that high collagen matrix concentration (2.0 mg/mL ≈ 17.9 kPa) could induce the p53 to activate the intrinsic apoptotic pathway through caspase 9 to generate smaller spheroids.

To better understand how the collagen matrix concentration and rigidity regulates EMT, we measured 3D matrix-dependent gene expression levels using RT-PCR (Figure 2J) and immunofluorescence (Figure 2I). We noticed that high collagen matrix concentration (2.0 mg/mL ≈ 17.9 kPa) upregulated E-cadherin gene expression while inducing significant downregulation of the mesenchymal markers, including vimentin, fibronectin, and Snail (Figure 2J). We also observed striking collagen matrix concentration/rigidity-dependent differences in E-cadherin, which showed prominent localization at cell-cell junctions in high collagen matrix concentration by confocal imaging (Figure 2I). Together, these data suggested that the high collagen matrix concentration and rigidity (2.0 mg/mL ≈ 17.9 kPa) triggered the epithelial properties (*i.e*, E-cadherin) but downregulated mesenchymal genes (*i.e*, N-cadherin, Vimentin and Snail).

Nevertheless, our results in Figures 1; Figure 2 demonstrated the spheroids size increment in low collagen matrix concentration may not be simply due to an increase in cell growth. Cells were seen to break away and spread from the main tumor spheroids (Figures 1A, E), resulting in the tumor mass becoming less compact (Figure 1D at the end of 5 days). This could be attributed to a low collagen matrix concentration, which in turn reduces the strength of cell-matrix adhesion. In addition, increasing collagen matrix concentration can also reduce matrix pore size, hence providing physical barriers to cell migration (Zaman et al., 2006). All these processes make it difficult to attribute the changes in spheroid growth to matrix concentration and rigidity alone, especially in the early stage of breast tumor growth.

### Spheroids’ growth is optimal in agarose of intermediate rigidity

Therefore, we selected agarose matrix as cells do not adhere directly to agarose and it cannot be remodeled or degraded by MMP. This ensures that the cells cannot migrate away from the tumor spheroid or alter the local rigidity of the ECM, making it a suitable matrix for studying the early stages of breast tumor growth, where cell-cell interactions play a more prominent role (Sant and Johnston, 2017). This also allows us to attribute any differences in spheroid growth to matrix concentration and rigidity.

To investigate the effects of agarose matrix rigidity on the cell growth of breast tumor spheroids, we embedded the spheroids in agarose matrices with rigidity levels of 0.6%, 1.0%, and 1.5%, corresponding to gel stiffness values of 8 kPa, 15 kPa, and 20 kPa, as previously characterized (Ahearne et al., 2005).

Achieving precise matching of stiffness values in collagen and agarose matrices presents a challenge due to inherent differences in the mechanical properties of these materials. Nevertheless, our aim was to uphold a comparable range of stiffness values in both matrices, ensuring a meaningful basis for comparison. The selected values (5.1 kPa, 11.4 kPa, 17.9 kPa in collagen; 8 kPa, 13 kPa, 20 kPa in agarose) were determined through careful consideration of the available literature and preliminary experiments. While not perfectly identical, these stiffness values fall within a biologically relevant range, maintaining a similar spectrum for both collagen and agarose matrices. By employing this comparable range of stiffness values, we intend to delve into matrix-specific effects and explore how matrix stiffness affects the responses of MCF7 spheroids.

**FIGURE 3:**
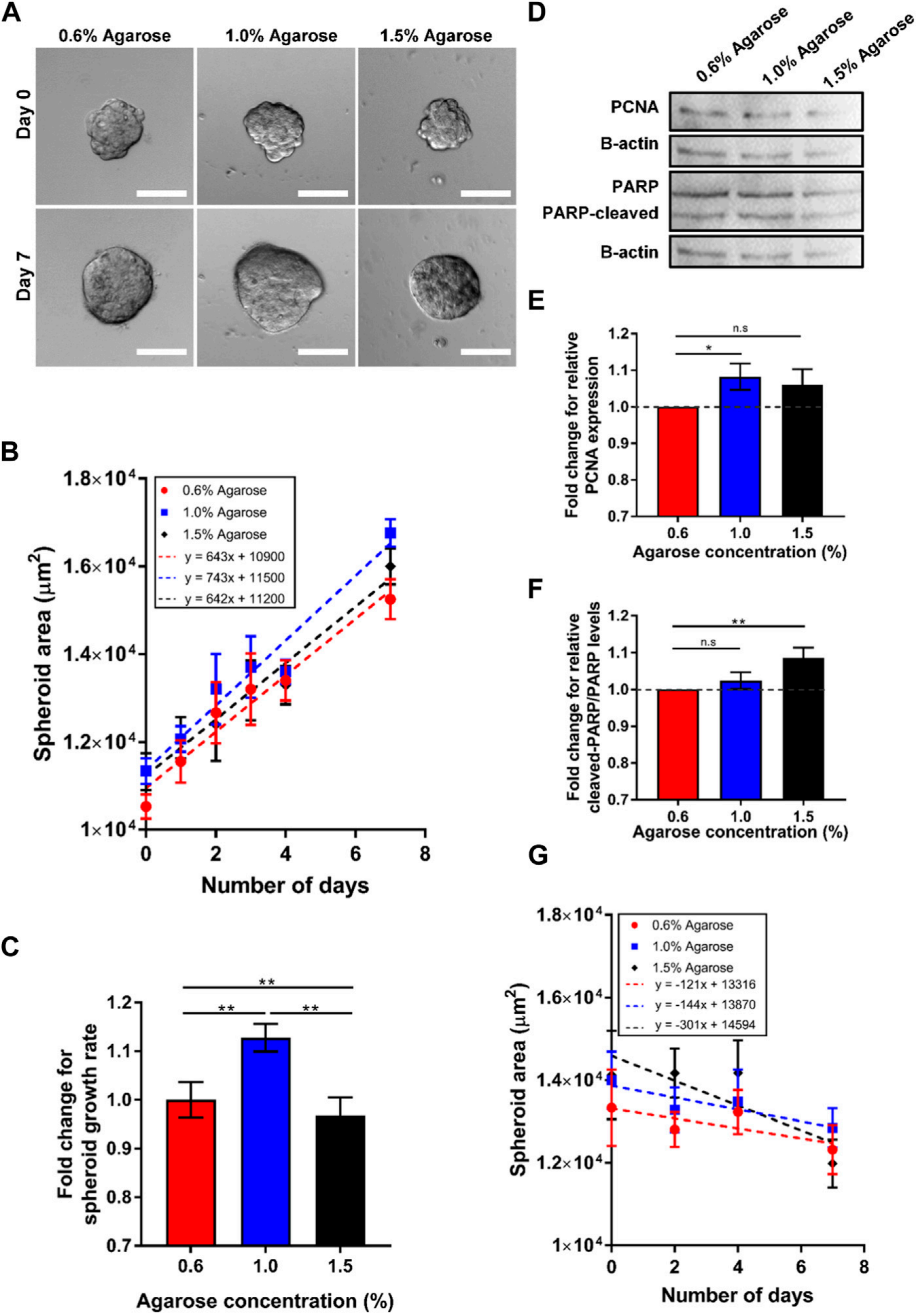
Spheroids embedded in 3D agarose matrices show optimal cell growth at intermediate concentration. **(A)** Brightfield images of spheroids embedded in 3D agarose gel of different concentrations on Day 0 and Day 7. Scale bar represents 100 μm. **(B)** Graph of spheroid area against the number of days the spheroids are embedded within the 3D agarose matrices (n = 84, 102, 51 for 0.6%, 1.0% and 1.5% agarose matrix concentrations respectively). **(C)** Fold change in spheroid growth rate for different agarose matrix concentration, normalized to the value for 0.6% agarose matrix concentration. **(D)** Western blot for the proliferation marker PCNA, the loading control b-actin, and the apoptosis marker PARP for spheroids embedded in agarose matrices of various concentrations. **(E)** Fold change in relative PCNA expression levels normalized to levels at 0.5% agarose matrix concentration (n = 5 independent experiments). **(F)** Fold change in relative ratio of cleaved PARP/PARP levels normalized to levels at 0.6% agarose matrix concentration (n = 5 independent experiments). **(G)** Graph of spheroid area against the number of days the spheroids are embedded within the 3D agarose gel with mitomycin c treatment (n = 24, 19, 14 for 0.6%, 1.0% and 1.5% agarose matrix concentrations respectively) Asterisks denote the statistically significant differences (student t-test, *p < 0.05; **p < 0.001; n.s = no significant differences).

To this end, the spheroids embedded in agarose were imaged over a 7-day period on a bright-field microscope (Figure 3A). The images were then segmented to obtain the projected spheroid area. The spheroid areas were found to increase over time for spheroids in all 3 agarose rigidities (Figure 3B). The growth of spheroid area vs. time fitted well to a linear relationship (*R* ^2^ = 0.959–0.969) and the gradients of the linear fits were obtained as the spheroid growth rate. The growth rate of the spheroids in the agarose of intermediate concentration (1.0% ≈ 15 kPa) was found to be the highest (743 μm^2^/day) as compared to the growth rate in the softest and stiffest agarose gel (643 μm^2^/day and 642 μm^2^/day for 8 kPa and 20 kPa gel rigidity respectively). The fold change of the spheroid growth rate at 1.0% (≈15 kPa) and 1.5% (≈20 kPa) agarose matrix concentration were found to be 1.13 and 0.967 times of the growth rate at 0.6% (≈8 kPa) (Figure 3C).

We hypothesize that the differences in spheroid area at various agarose matrices concentration and rigidity arise due to differences in cell growth and apoptosis rate of the spheroids. Western blot for the expression of a common proliferation marker protein, PCNA, also showed the highest expression of PCNA at intermediate agarose matrix concentration with a fold change of 1.06 with respect to the amount of PCNA at 0.6% agarose matrix (≈8 kPa) (Figures 3D,E). In contrast, the fold change of PCNA expression for spheroids embedded within the 1.5% agarose matrix (≈20 kPa) was found to be lower, at 0.989. To quantify the rate of apoptosis, the presence of cleaved- Poly (ADP-ribose) polymerase (PARP), which serves as a marker of apoptosis, could be quantified. Western blot analysis for PARP showed that the ratio of cleaved-PARP to PARP was increased by 1.02 and 1.06-fold as agarose matrix concentration is increased to 1.0% (≈5 kPa) and 1.5% (≈20 kPa) respectively, as compared to the expression levels at 0.6% (≈8 kPa) (Figures 3D, F). In addition, when cell growth was blocked by the cell cycle inhibitor mitomycin c, spheroid growth rates were consistently negative at all agarose matrix concentration, with the growth rate becoming increasingly negative (−121 to −301 μm^2^/day) as agarose matrix concentration increased from 0.6% (≈8 kPa) to 1.5% (≈20 kPa) (Figure 3G). These results suggest that increasing agarose matrix concentration and rigidity results in higher rate of cell apoptosis and the optimal growth rate at intermediate agarose matrix concentration of 1.0% (≈15 kPa) arise primary due to a higher cell growth rate at the intermediate agarose matrix concentration.

### 3D force measurements reveal 2 distinct population of cells in growing tumor spheroids

**FIGURE 4:**
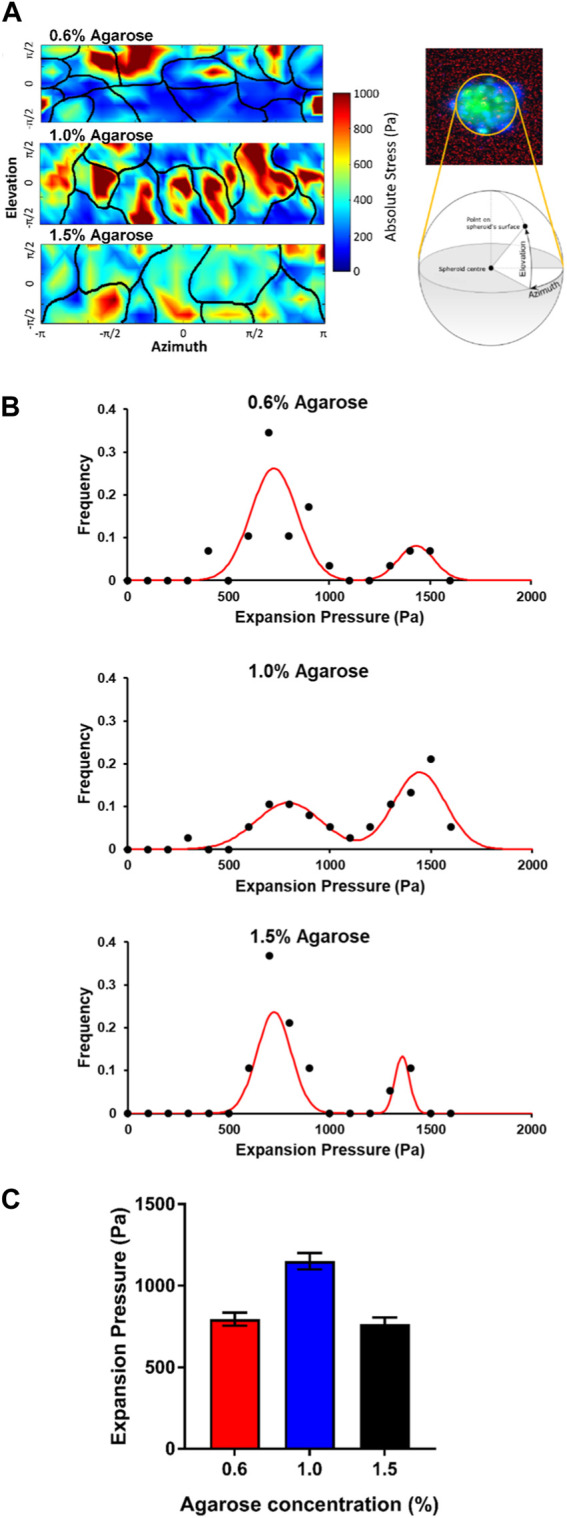
Cells on the surface of the embedded spheroids exert expansion forces on the agarose matrices **(A)** Expansion stress maps of stresses exerted on the agarose matrix by cells at the spheroid surface at various elevation and azimuth of the spheroid surface. Black lines illustrate the cell-cell boundaries. **(B)** Graphs showing the frequency of cell expansion pressures exerted by each cell on the spheroid-agarose interface at different agarose matrix concentrations. **(C)** Plot of average expansion pressure exerted by the spheroids vs. agarose matrix concentrations.

It is known that in a multicellular spheroid, the proliferating cells are largely located at the periphery of the spheroid, with the middle layer of cells in the spheroid comprising of viable but quiescent cells, surrounding a central necrotic core for spheroids larger than 200 µm (Gong et al., 2015). As the proliferating cells round and divide, the cells exert a rounding pressure against the ECM (Stewart et al., 2011). We therefore wondered if these rounding pressures can be detected by the deformation of the agarose gels. Agarose is an elastic matrix which cannot be remodeled or degraded by MMP, hence making it a suitable material to measure forces exerted by the growing spheroids on the ECM. Fluorescent beads were embedded within the agarose gels as markers to detect any gel deformation induced by the spheroids, and imaged at 30 min interval to obtain the difference in gel deformation due to spheroid expansion between the 2 time-frames. The movement of the embedded beads was used to calculate the gel strain, from which an expansion stress on the surface of the spheroids can be obtained (Figure 4A). Each of the stress vector can then be mapped to the closest cell at the periphery of the spheroid.

Interestingly, for all agarose matrix concentrations, we were able to group cells into 2 populations based on the magnitudes of the expansion stress measured. The first population of cells exerted between 500 and 1,000 Pa of stress on the agarose matrices while the second population of cells exerted between 1,200 and 1,600 Pa of stress on the agarose matrices (Figure 4B). We postulate that the two population of cells which exerted low and high expansion stress may be attributed to non-dividing and dividing cells respectively.

For the spheroids embedded in the 0.6% (≈8 kPa) and 1.5% (≈20 kPa) agarose gels, we observed that a greater proportion of the cells belong to the first population which exerted low expansion stress of 500–1,000 Pa (75.9% and 78.9% for 0.6% (≈8 kPa) and 1.5% (≈20 kPa) respectively) at the spheroid-gel boundary. On the other hand, a much lower proportion of cells in the spheroids embedded in the 1.0% (≈15 kPa) agarose matrix fall into this first population that exerted low expansion stress (39.4%) at the spheroid-gel boundary. Instead, most of the cells in the spheroids embedded in the 1.0% (≈15 kPa) agarose matrix belong to the second population of cells which exerted a high expansion stress of 1,200–1,600 Pa (55.2%) at the spheroid-gel boundary. In comparison, only 17.2% and 15.8% of cells in the spheroids embedded in 0.6% (≈8 kPa) and 1.5% (≈20 kPa) respectively belong to this second population of cells which exerted high expansion stress at the spheroid-gel boundary. As a result, the average expansion pressure is the highest for spheroids embedded in the agarose gel of intermediate rigidity 1.0% (≈15 kPa) (Average expansion stress = 795, 1,150 and 765 Pa for agarose matrix concentration = 0.6%, 1.0% and 1.5% respectively; Figure 4C). Taken together, the expansion pressure results provide support that spheroids are expanding and proliferating more when embedded in agarose gels of intermediate matrix concentration (1.0% agarose ≈15 kPa).

### Cell rounding pressure during mitosis determines optimum matrix concentration for spheroid growth

Previous studies have shown that during mitosis, the cell rounding pressure is regulated by the cell actomyosin cortical tension and the cell hydrostatic pressure (Stewart et al., 2011). By adding inhibitors or activators of myosin activity, the actomyosin cortical tension and hence the cell rounding pressure, can be reduced or increased respectively (Ramanathan et al., 2015; Cartagena-Rivera et al., 2016). It has been previously shown that mitotic cells, which cannot generate a sufficient outward directed force to round up against mechanical confinement in their environment, were less viable and more likely to undergo apoptosis (Sorce et al., 2015). We therefore hypothesize that the optimum cell growth rate of the spheroids at intermediate matrix concentration (1.0% agarose ≈15 kPa) could be attributed to the actomyosin cortical tension. We perturbed the actomyosin cortical tension by treating the spheroids with the myosin inhibitor, blebbistatin, and the myosin activator, calyculin A, to reduce and increase the cortical tension respectively (Ramanathan et al., 2015; Cartagena-Rivera et al., 2016).

**FIGURE 5:**
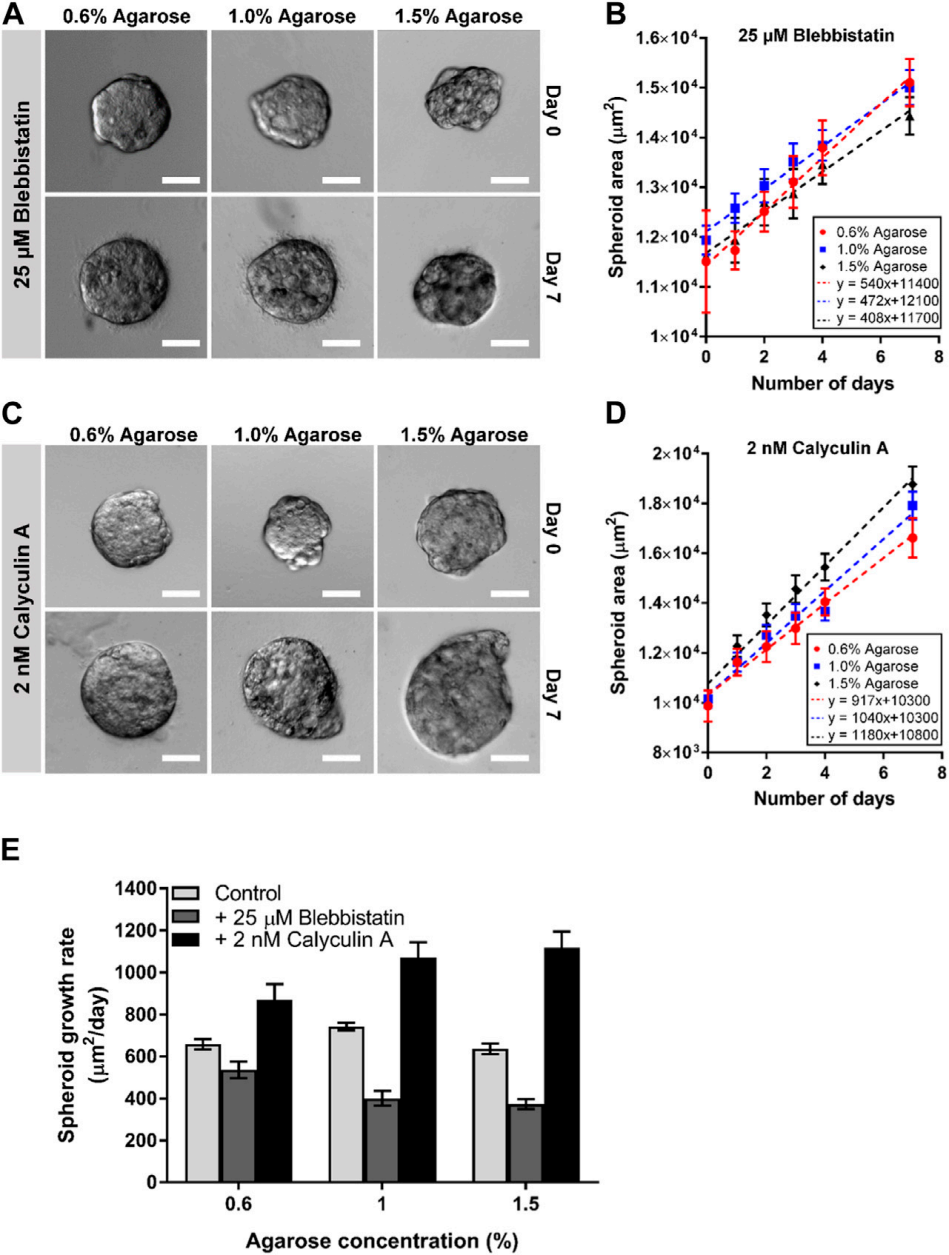
Perturbing actomyosin contractility of spheroids embedded in 3D agarose matrices alters spheroid growth. **(A)** Brightfield images of spheroids embedded in 3D agarose matrix of different concentrations on Day 0 and Day 7 upon treatment with 25 μM blebbistatin. Scale bar represents 100 μm. **(B)** Graph of spheroid area against the number of days the spheroids are embedded within the 3D agarose matrix with 25 μM blebbistatin treatment (n = 6 19, 20, 21 for 0.6%, 1.0% and 1.5% agarose matrix concentrations respectively). **(C)** Brightfield images of spheroids embedded in 3D agarose matrix of different concentrations on Day 0 and Day 7 upon treatment with 2 nM calyculin **(A)**. Scale bar represents 100 μm. **(D)** Graph of spheroid area against the number of days the spheroids are embedded within the 3D agarose matrix with 2 nM calyculin A treatment (n = 19, 22, 20 for 0.6%, 1.0% and 1.5% agarose matrix concentrations respectively). **(E)** Spheroid growth for different agarose matrix concentrations with the various drug treatment.

We observed that reducing the cells actomyosin cortical tension through blebbistatin generally reduced the spheroid growth rate as compared to the untreated spheroids (minimum-maximum growth rate for blebbistatin: 408–540 μm^2^/day, and untreated: 643–743 μm^2^/day; Figure 5E). We also observed that the blebbistatin-treated spheroid growth rate reduces monotonically as agarose matrix concentration was increased from 0.6% to 1.5% (≈8–22 kPa) (540–408 μm^2^/day; Figures 5A, B, E). Interestingly, the addition of calyculin A to increase actomyosin cortical tension was found to generally increase spheroid growth rate as compared to the untreated spheroids (minimum-maximum growth rate for calyculin A: 917–1,180 μm^2^/day, and untreated: 643–743 μm^2^/day; Figure 5E). The calyculin A-treated spheroids also showed an increasing growth rate as agarose matrix concentration is increased (917–1,180 μm^2^/day; Figures 5C–E). These results suggest that the optimal matrix concentration for spheroid growth could be related to the strength of the cells actomyosin cortical tension.

## Discussion

During breast tumor progression, it has been shown that ECM rigidity increases due to increased collagen deposition (Provenzano et al., 2008) and tumor cells in the growing tumor spheroid have to proliferate and expand against increasing mechanical stress imposed by the stiffness of the ECM (Montel et al., 2012). A mechanistic understanding of tumor spheroid growth in response to mechanical rigidity due to the matrix concentration of the 3D ECM is however lacking. Ondeck et al. (2019) demonstrated that breast cancer spheroids remained spherical in hydrogels with rigidity of 0.1 kPa–3 kPa, and the tumor spheroids started to spread on hydrogels with rigidity of 3 kPa–5 kPa due to the EMT process (Ondeck et al., 2019). Another interesting study from Taubenberger et al. (2019) showed that MCF7 spheroids appeared smaller and circular in hydrogels with rigidity of ≈20 kPa due to the increasing compression force of the stiff hydrogel in decreasing their growth (Taubenberger et al., 2019). Similarly, Charoen et al. (2014) demonstrated that the diameter of breast cancer spheroids embedded in collagen gels were getting smaller in increasing collagen concentration from 2 mg/mL to 5 mg/mL (>20 kPa) (Charoen et al., 2014). Taken together, these studies revealed that the spheroids remained circular and smaller either in very low rigidity hydrogel (0.1–3 kPa) or in very high rigidity hydrogel (>20 kPa) but the optimal cell growth of the breast cancer spheroids remain unexplored. To fill the gap, we are motivated to discover the spheroid growth profile and explore the underlying mechanisms to determine if there exists an optimal range of cell growth within the rigidity spectrum of approximately 5 kPa (low rigidity) to 20 kPa (high rigidity) by varying the matrix concentration. This range aligns with the reported stiffness range of human breast cancer tissue from biopsies, which spans from 0.3 kPa to 20 kPa (Plodinec et al., 2012; Acerbi et al., 2015), Given that collagen is a physiologically-relevant matrix, we first embedded the MCF7 spheroids in collagen matrices with different concentrations which contributed to different rigidities. We have shown that for MCF7 tumor spheroids, the spheroid growth is the lowest at the highest collagen matrix concentration (2.0 mg/mL ≈ 17.9 kPa) (Figure 1). However, we also observed that substantial cell detachment and migration away from tumor spheroids at the lowest collagen matrix concentration (0.5 mg/mL ≈ 5.1 kPa) but not at the highest collagen matrix concentration (2.0 mg/mL ≈ 17.9 kPa), as illustrated in Figure 2A (arrows). Furthermore, the spheroids also appeared to spread away from the tumor mass, presenting a less compact structure following a 5-day culture within the 3D collagen matrix. Our observations align with Mahajan and colleagues’ study, where increased stiffness of degradable hydrogel (15–20 kPa) similarly reduces the growth of MCF7 spheroids, accompanied by lower invasion into the matrix (Mahajan et al., 2021). Our further investigation revealed that changes in spheroid sizes and morphologies appeared to be regulated by cell growth, cell cycle arrest, apoptosis, and EMT when embedded in collagen matrices. Our findings correlate with the study of Geiger et al. (2022), demonstrating that tumor spheroids within 3D collagen matrices revealed invasion capabilities due to EMT (Geiger et al., 2022). In addition, they also illustrated that cancerous samples exhibit fluidity due to active cell movement within the tissue. Nevertheless, it is difficult to attribute the optimal cell growth observed at low collagen matrix concentration to the changes in matrix rigidity alone, not only because of the EMT, but also the changing collagen matrix concentration also introduces changes in matrix rigidity, porosity, cell-matrix adhesion and cell migration away from the spheroid (Zaman et al., 2006).

Therefore, we sought to establish the role of matrix concentration and rigidity alone on spheroid growth, by embedding the MCF7 spheroids in agarose matrices with various agarose concentrations. As the cells cannot directly adhere to agarose (Sant and Johnston, 2017), increasing agarose gel concentrations will only increase ECM rigidities without altering cell-matrix adhesion, matrix porosity or rate of cell migration away from the spheroids. Interestingly, we observed the fastest spheroid growth at an intermediate agarose matrix concentration (1.0% ≈ 15 kPa) (Figure 3C), thus supporting the hypothesis that the optimal spheroid growth can be attributed to an optimum mechanical rigidity of the ECM when spheroids are confined within the 3D matrix. The increase in spheroid size is mainly due to cell growth as inhibition of cell growth through the use of the cell division inhibitor mitomycin c (Shatkin et al., 1962) decreased spheroid size over time (Figure 3G). This rate of decrease in spheroid size increases as agarose matrix concentration and rigidity increases, suggesting that increasing ECM concentration and rigidity increases cell apoptosis rate. This is in agreement with earlier studies which have found increasing mechanical confinement and compression on tumor spheroids reduces spheroid growth rate. For example, Delarue et al. (2014) and Montel et al. (2012) have shown that mechanically compressing spheroids with Dextran in the cell culture media increases the expression of the cell proliferation inhibitor p27^Kip1^, bringing about a reduction in the spheroids’ growth rate (Montel et al., 2012; Delarue et al., 2014). Desmaison et al. (2018) have also shown that physically confining tumor spheroids in agarose gels induced prometaphase delay as compared to freely growing spheroids (Desmaison et al., 2018). We have also verified that PARP, which is involved in DNA repair and inactivated by caspase cleavage during apoptosis (Morales et al., 2014), is increasingly cleaved as matrix rigidity is increased (Figures 3D, F), providing evidence that increasing matrix rigidity increases rate of cell apoptosis.

In this study, we selected different apoptosis indicators, caspases 8, 9, 3, 7 for collagen-embedded spheroids and cleaved-PARP for agarose-embedded spheroids, due to the distinct properties of the embedding matrices and their potential influence on cellular responses. In collagen-embedded spheroids, where tumor cells adhere and interact with the collagen, there are multifaceted effects on cellular processes such as proliferation, apoptosis, and EMT, effectively mimicking the tumor progression. Given the importance of understanding apoptosis in this context, where cellular interactions and matrix adhesion play pivotal roles, we opted for measuring caspase activities (3, 7, 8, 9). This decision allows for a more specific and detailed assessment of the apoptotic cascade, providing insights into the dynamic aspects of cell death pathways. In contrast, agarose-embedded spheroids were chosen to simulate the early stages of breast tumor growth, where cell-cell interactions are more pronounced and matrix adhesion is minimal. In this setting, our focus shifted towards understanding cell-cell interactions rather than the caspase cascade. To study apoptosis in this context, previous studies have suggested that monitoring cleaved-PARP in agarose-embedded systems provides valuable insights into the downstream events of apoptosis. Therefore, we employed cleaved-PARP as an apoptosis marker for agarose-embedded spheroids.

While rate of cell apoptosis increases when matrix concentration and rigidity is increased, cell growth rate displays a biphasic relation with matrix rigidity. We have shown that the fastest rate of cell growth occurs at the intermediate matrix concentration of 1.0% agarose (≈15 kPa), as revealed by the presence of the highest amount of proliferation marker PCNA at the intermediate rigidity (Figures 3D, E). As the spheroid grow and expand against the mechanical confinement imposed by the agarose gels, the agarose gels will deform and the expansion stress exerted by the spheroid on the gel can be quantified based on the displacements of fluorescent beads embedded within the gels. We have quantified the expansion stress of the growing tumor spheroid and showed that at the intermediate matrix concentration of 1.0% agarose (≈15 kPa), the average expansion stress exerted at the spheroid-gel boundary is the highest among the 3 agarose matrix concentrations (Figure 4C). The expansion pressures observed at the spheroid-gel boundaries (≈800–1,200 Pa) are also comparable with the mechanical stress measured on the surface of tumor spheroids (≈1000Pa) quantified by Dolega et al. (2017) under 5 kPa of mechanical compression (Dolega et al., 2017). In order to measure changes in mechanical stress propagation within a tumor spheroid, Dolega et al. (2017) embedded deformable polyacrylamide beads of well-defined elasticity in the tumor spheroids and subjected the spheroids to mechanical compression induced by adding Dextran into the culture media. With this method, mechanical stress at different depths within the tumor spheroid could be measured based on the deformation of the polyacrylamide beads. However, this method faces the limitation that stresses can only be quantified at discrete regions in proximity of the polyacrylamide beads. In contrast, while our expansion stress measurements can only detect stresses exerted at the spheroid-gel interface, we were able to assign each stress to individual cells on the spheroid surface, thus offering stress measurements at single cell resolution.

In a separate study, Gordon et al. (2003) embedded tumor spheroids within matrigel with fluorescent beads embedded to track gel displacements as the spheroid expands (Gordon et al., 2003). However, the authors noted that the cells adhered to the matrix and invaded into the matrigel, remodeling and possibly altering the mechanical properties of the matrigel in the process. All these made characterizing the expanding pressures on the surface of the tumor spheroid challenging. By embedding the tumor spheroids within deformable agarose gels, where cells were unable to adhere and migrate away from the spheroids, we were able to characterize spheroid expansion stress at the spheroid-gel boundaries at single cell resolution.

Fuhs et al. (2022) suggested that primary breast tumor explants from patients consist of two distinct groups, which are soft and rigid cancer cells, resulting in regions characterized by rigid cells surrounded by soft and motile cells (Fuhs et al., 2022). Interestingly, Grosser and colleagues also identified two active states in solid tumors: one being a disordered fluid (unjammed) state, and the other characterized as an amorphous glasslike (jammed) state (Grosser et al., 2021). Building upon this, our investigation revealed, based on measured expansion stress, cells can also be classified into two groups, one exerting a lower expansion stress (500–1,000 Pa) and the other exerting a higher expansion stress (1,200–1,600 Pa). We postulated that the two populations of cells which exerted low and high expansion stress may be attributed to non-dividing and dividing cells, respectively. This is because a larger population of the cells on the tumor spheroid surface exerted high expansion stress of between 1,200 and 1,600 Pa in the 1.0% agarose matrix (≈15 kPa) (Figure 4B), correlated with the observation of higher expression of the proliferation marker in spheroids embedded in the 1.0% agarose matrix (≈15 kPa) as compared to the other agarose matrix concentrations and rigidities.

Our identification of cells exerting low (500–1,000 Pa) and high (1,200–1,600 Pa) expansion stress levels aligns conceptually with the two states identified by Grosser et al. (2021) and Fuhs et al. (2022), as we postulate that these populations may correspond to non-dividing and dividing cells, or rigid and soft cells, respectively. Moreover, our observation that agarose stiffness influences the distribution of these populations, particularly in softer gels, correlates with Grosser *et al.*'s emphasis on cell fluidity and migratory activity on the spheroid surface as potential indicators of metastatic potential (Grosser et al., 2021). Together, our studies contribute to a more comprehensive understanding of the dynamic interplay between matrix properties, cellular behaviors, and their implications for cancer progression.

In addition, Stewart et al. (2011) have shown that during cell division, the mitotic cell rounds up and exert a mitotic rounding pressure, which is about 3-folds higher than non-dividing pre-rounded cells in G2/S phase (Stewart et al., 2011). However, the rounding pressure was found to be about 150 Pa which is much lower than the expansion stress we have characterized in our study. The difference may be attributed to differences in the experimental design: the rounding pressure characterized by Stewart et al. (2011) was done on single HeLa cells grown on a flat 2D glass-bottomed dish while our expansion pressure was characterized on MCF7 cells in a multicellular system as a tumor spheroid embedded in a soft 3D matrix.

Cattin et al. (2015) have applied mechanical forces using microcantilevers to confine single cells undergoing mitosis and characterize their progressions through mitosis (Cattin et al., 2015). It was found that as compared to unconfined cells, an application of a small 5 nN force accelerated mitotic progression, possibly by aligning the cells’ long axis parallel to the substrate or providing a slight tension on the mitotic spindle. However, the authors also found that application of a force greater than 50 nN slowed and even stopped mitosis as cell rounding and mitotic spindle assembly is impaired, leading to failure of cells to initiate chromosome segregation. We therefore hypothesize that during cell division, the dividing cells on the surface of the spheroid round up and push against the confining agarose matrices, exerting an expansion pressure on the surrounding agarose gel. As the agarose matrix concentration and rigidity increases, the agarose matrix also exerts increasing opposing mechanical stress on the dividing cells, which can accelerate mitotic progression. However, beyond a certain threshold, if the matrix concentration and rigidity is too high, the dividing cells may not be able to deform the matrix sufficiently during cell rounding, thus inhibiting mitotic progression. Therefore, an optimum matrix concentration and rigidity exists to regulate cell mitosis, depending on the mitotic rounding pressure generated by the dividing cell. Stewart et al. (2011) have shown that the magnitude of the mitotic rounding force is dependent on the cell’s osmotic pressure and the actomyosin cortical tension (Stewart et al., 2011). By perturbing actomyosin cortical tension through the use of blebbistatin or calyculin A, which decreases or increases actomyosin tension respectively (Ramanathan et al., 2015; Cartagena-Rivera et al., 2016), we observed that the optimal matrix concentration and rigidity for fastest spheroid growth decreased and increased respectively. The results suggest that for spheroids which are confined growth in a 3D matrix, the actomyosin cortical tension plays a critical role in influencing spheroids and the matrix concentration and rigidity at which maximal spheroid growth occurs.

In the 3D collagen matrices, cells adhere to the collagen to initiate EMT, prompting them to migrate outward from the spheroids. In this context, actomyosin activity, primarily associated with cellular contraction, may not play a pivotal role. The adherence and migration dynamics of cells are more influenced by interactions with the collagen matrix and EMT processes, rendering actomyosin-driven forces less crucial. Therefore, we did not study actomyosin activity in collagen hydrogels, where its impact on spheroid growth appeared to be less influential. In contrast, the MCF7 tumor cells do not adhere to agarose matrices, but instead rely more on cell-cell interactions. Therefore, actomyosin activity becomes more relevant, especially in the study of expansion force.

## Conclusion

In conclusion, we have found that MCF7 tumor spheroid growth is the fastest at the lowest collagen matrix concentration and rigidity (0.5 mg/mL ≈ 5.1 kPa) and the intermediate agarose matrix concentration and rigidity of 1.0% agarose ≈11.4 kPa. This discrepancy was probably due to the remodeling of the collagen matrix which allowed the cell-matrix adhesion and migration from tumor spheroids through EMT, leading to changes in collagen matrix mechanical properties. We then focused our studies on spheroids embedded within agarose matrices as cells do not adhere directly to agarose to prevent ECM degradation through EMT, to attribute the changes in spheroid growth to matrix concentration and rigidity alone. We also developed an algorithm to characterize the stresses exerted by the expanding spheroid on the agarose matrix. We found that cells on the spheroid surface can be classified into 2 distinct populations, one generating a lower expansion force of approximately 500–1,000 Pa and the other exerting a higher expansion force of approximately 1,200–1,600 Pa, possibly arising from non-dividing and dividing cells respectively. In accordance with the optimum cell growth at intermediate matrix concentration and rigidity, we also observed a higher proportion of cells exerting high expansion stress in the spheroids embedded in intermediate agarose matrix concentration, suggesting the presence of more dividing cells in these spheroids in the intermediate agarose matrix concentration. This hypothesis agrees with higher expression levels of the cell proliferation marker PCNA in spheroids grown in the intermediate agarose matrix concentration. Treatment of the spheroids with an inhibitor and activator of myosin activity, which increase or decrease cell cortical tension respectively, showed that the matrix concentration and rigidity for optimum spheroid growth can be decreased or increased respectively. These results indicate that the optimal matrix concentration, leading to maximal spheroid growth, is determined by the cell’s cortical tension, which in turn influences the pressure exerted during cell division and the process of cell rounding.

## Methods and materials

### Cell culture

MCF7 human breast adenocarcinoma cells were maintained in high glucose (4.5 mg/mL) Dulbecco’s modified Eagle’s medium (Life Technologies, Carlsbad, CA) supplemented with 10% fetal bovine serum (Life Technologies) and 1% penicillin-streptomycin (Life Technologies) at 37°C, 5% CO_2_, and 100% humidity.

### 3D culture of spheroids in collagen and agarose matrices

To generate MCF7 spheroids for embedding in collagen or agarose gels, 2 × 10^6^ cells were seeded onto a non-adherent 90 mm diameter Petri dish for 3 days. The aggregated cell spheroids were then filtered with a 100 µm and a 70 μm cell strainer (Fisher Scientific) to obtain spheroids between the size of 70–100 µm diameter.

To embed the 3D spheroids in collagen type I gel, a “sandwich” method as described in Arytm and Matsumoto (Artym and Matsumoto, 2010) was employed. In brief, 3 mg/mL collagen type 1 solution (Thermo Fisher) was neutralized with NaOH and diluted in 10 x PBS according to the manufacturer’s specification. Final concentration of the collagen solution was prepared to 0.5 mg/mL, 1.0 mg/mL or 2.0 mg/mL which approximates to the stiffness of 5.1 kPa, 11.4 kPa and 17.9 kPa respectively (Provenzano et al., 2009). 200 μL of the pre-polymerized collagen gel was first added to each 24-well plate. To promote polymerization, the culture dish was incubated in a 37°C incubator for 1 h. After the first layer of collagen has gelled, the second layer of the collagen-spheroid mixture was added and further incubated in a 37°C incubator for 1 h. Thereafter, 500 uL of cell culture media was added into each well and the spheroid-collagen culture was kept for 5 days with a media change on day 3.

To embed the spheroids in agarose gel, spheroids suspended in cell culture media are mixed with low melting point agarose such that the final concentration of the agarose is either 0.6%, 1.0%, or 1.5% to give agarose gels of rigidity 8 kPa, 15 kPa and 20 kPa respectively (Ahearne et al., 2005). 300 μL of the spheroid-agarose mixture was added to each well of the 12-well plate and placed on top of ice for 5 min to allow the agarose to form gel. Thereafter, 1 mL of cell culture media was added into each well and the spheroids were allowed to grow in the agarose gel for 7 days with a media change on day 3.

### Tracking spheroid growth

Phase-contrast images of live MCF7 spheroids embedded in both 3D collagen matrix and agarose were acquired using an inverted fluorescence microscope (Axio Observer Z1; Zeiss, Oberkochen, Germany) equipped with a ×20 objective (0.8 NA), with a scientific charge-coupled-device (CCD) camera (Cool SNAP HQ2; Photometrics, Tuscon, USA) every 24 h for the duration of the experiment to track the spheroid growth. Images analysis algorithm was developed using Python to calculate the area and circularity (defined as 4×π×area/(perimeter)^2^) of the spheroids. The fold change for the spheroid growth rate was calculated by establishing ratios between the slope coefficients of different stiffness conditions against the reference point, which is the softest stiffness.

### Immunofluorescence staining, imaging and quantification

At day 5, the spheroids embedded in 3D collagen matrix were fixed in 3.7% paraformaldehyde at room temperature for 1 h, followed by an overnight permeabilization step in 1% Triton X-100 solution at 4°C on an orbital shaker. After permeabilization, spheroids were incubated with primary antibodies diluted in antibody dilution buffer consisted of 2% BSA, 0.2% Triton-X 100 in 1 x PBS at 4°C for 48 h. The primary antibodies used in this study including Ki-67(Rabbit, MA514520, Invitrogen, 1:50), Caspase 3 (rabbit, 700,182, Invitrogen, 1:50), Anti E-cadherin (Rabbit, MA514458, Invitrogen, 1:50) and Anti-Vimentin (mouse, MA511883, Invitrogen, 1:50). The samples were then rinsed with washing buffer (3% NaCl and 0.2% Triton-X 100 in 1 x PBS) for 1 h at room temperature twice followed by overnight incubation in washing buffer at 4°C on an orbital shaker. Then, the samples were stained with secondary antibodies, 0.5 μg/mL DAPI (Invitrogen), and Alexa Fluor 488 phalloidin (A12379, Invitrogen, 1:200) (if required) at 4°C on an orbital shaker overnight. The secondary antibodies used in this study were donkey anti-rabbit IgG-Alexa Fluor 488 (Invitrogen, 1:400), donkey anti-rabbit IgG-Alexa Fluor 647 (Invitrogen, 1:400), donkey anti-mouse IgG-Alexa Fluor 555 (Invitrogen, 1:400). After overnight incubation, the samples were then rinsed according to the similar washing steps as after primary antibody incubation. The samples were placed in 0.5 mm deep spacers with mounting media (Vectashield), followed by sealing with a coverslip. The immunofluorescence images were acquired using Nikon A1Rsi DUVB Confocal laser scanning system (Nikon) with a ×40 objective. Z-stacks at intervals of 1 μm, were obtained to enable the calculation of spheroids circularity, volume and the nuclei in three-dimensional (3D) spheroids. To analyze spheroids circularity, immunofluorescence images of F-actin were used. These fluorescent images were processed and analyzed by employing a Python algorithm developed in-house. In brief, the enhanced local contrast was first applied followed by segmenting the F-actin using a threshold value determined through the Yen method. Subsequent binary operations, including closing, removing small objects, filling holes, and opening, were applied to obtain the segmented cell area. Following this, the circularity values were determined using the formula 4×π×[area/(perimeter^2^)] (Supplementary Figure S1). Spheroids volume was the sum of all the volumes (area x 1 μm (resolution of the z-stack)) of spheroids in each stack. The quantification of nuclei in 3D spheroids for DAPI, Ki-67, and caspase 3 analysis was done using our dictionary-based denoising method to segment, detect, and calculate the total cell nuclei in 3D stacked images of a spheroid (Nasser and Boudier, 2019).

### RNA isolation and qRT-PCR on tumor spheroids in collagen gels

The spheroids embedded in the collagen matrix were lysed using Trizol Reagent (Life Technologies). Then, the RNA was isolated from samples using RNeasy Micro kits (Qiagen) according to the manufactural protocols. Subsequent RNA concentration was then measured with a NanoDrop (Thermo Scientific). Reverse transcription to cDNA was performed using Tetro cDNA Synthesis Kit (Bioline). Real-time quantitative PCR was performed with FastStart universal SYBR Green Master reagents (Rox) (Roche) in a ViiA 7 Real-Time PCR System (Thermo Fisher Scientific). Fold changes of transcripts in D5-cultured MCF7 spheroids embedding in collagen matrix relative to D0-cultured MCF7 free spheroids were determined by ΔΔCt to study the gene expression of growth, cell cycle arrest, apoptosis, and EMT. The primers sequences used in this study are listed in Table 1.

**TABLE 1:** Primers used for MCF7 spheroids embedded in collagen gel.

Primer	Forward	Reverse
GAPDH	GAG​TCA​ACG​GAT​TTG​GTC​GT	GAC​AAG​CTT​CCC​GTT​CTC​AG
Ki-67	TCC​TTT​GGT​GGG​CAC​CTA​AGA​CCT​G	TGA​TGG​TTG​AGG​TCG​TTC​CTT​GAT​G
PCNA	CCA​TCC​TCA​AGA​AGG​TGT​TGG	GTG​TCC​CAT​ATC​CGC​AAT​TTT​AT
P53	GCC​CAA​CAA​CAC​CAG​CTC​CT	CCT​GGG​CAT​CCT​TGA​GTT​CC
P21	AAGACCATGTGGACCTGT	GGT​AGA​AAT​CTG​TCA​TGC​TG
GADD45	GCA​ATA​TGA​CTT​TGG​AGG​AAT​TC	CCA​TCA​CCG​TTC​AGG​GAG​ATT
Caspase 8	CCA​GAG​ACT​CCA​GGA​AAA​GAG​A	GAT​AGA​GCA​TGA​CCC​TGT​AGG
Caspase 9	ATT​GCA​CAG​CAC​GTT​CAC​AC	TAT​CCC​ATC​CCA​GGA​AGG​CA
Caspase 3	ATG​CAT​ACT​CCA​CAG​CAC​C	ACC​ACC​AAC​CAA​CCA​TTT​C
Caspase 7	TCT​GGT​GCT​GTC​TTT​TGT​TCT​C	TTC​TTG​CTG​TTT​GGC​TTC​TCT
E-cadherin	TTGACGCCGAGAGCTACA	GAC​CGG​TGC​AAT​CTT​CAA​A
N-cadherin	ACC​AGG​TTT​GGA​ATG​GGA​CAG	ATG​TTG​GGT​GAA​GGG​GTG​CTT​G
Vimentin	TGA​AGG​AGG​AAA​TGG​CTC​GTC	GTT​TGG​AAG​AGG​CAG​AGA​AAT​CC
Snail	ATC​GGA​AGC​CTA​ACT​ACA​GCG​AGC	CAG​AGT​CCC​AGA​TGA​GCA​TTG​G

### Quantification of apoptosis using caspase assay

To examine the active caspase activities, MCF7 spheroids were cultured in 96-well plate after embedding to different collagen concentrations. After 5-day of culture, the Caspase-Glo reagents (Promega) including Caspase-Glo 3/7, 8, and 9 assay reagents were added directly to individual wells in 96-well plates on an orbital shaker for 30s, and further incubated at 37°C in 5% CO_2_ for 30–60 min prior to luminescence reading. Luminescence measurements were obtained using the microplate reader Gen 5TM (BIO-TEK Instrument, Vermont, USA) to reflect the active caspase activities.

### 3D expansion stress for agarose-spheroids

For 3D expansion stress measurements (Figure 4A), the spheroids’ cytoplasm were stained with CellTracker Green CMFDA Dye (Life Technologies) for 1 h prior to mixing with the agarose. 80 μL of 0.5 µm diameter red fluorescent beads (Life Technologies) were also added into 1 mL of the spheroid-agarose mixture and mixed thoroughly before adding 100 µL of the spheroid-bead-agarose mixture to a 35 mm diameter glass-bottom dish (Mattek). The glass-bottom dish was then placed on top of ice for 5 min to allow the agarose to gel, after which the dish was top up with 1 mL of cell culture media, and imaged 3 days later. Prior to imaging, the spheroids embedded in the agarose gel were stained for the nuclei using a final concentration of 1 μg/mL Hoechst 34,580 (Life Technologies). 3D confocal image stacks of the spheroids and the fluorescent beads embedded in the agar were obtained at an interval of 30 min for calculation of the spheroid expansion stress.

### Drug treatments

For spheroids treated with mitomyosin C, blebbistatin or calyculin A, final concentrations of 5 μg/mL mitomycin c (Sigma), 50 µM blebbistatin (Tocris Bioscience, Bristol, United Kingdom), or 2 nM calyculin A (Sigma St. Louis, MO) were added to the cell culture media and replaced every 3 days.

### SDS-PAGE

For spheroids grown in agarose gels, spheroids were grown for 7 days and the spheroid-agarose gels were washed in cold PBS twice prior to homogenization and solubilization with cold RIPA buffer (Pierce) and 22G needle for 30 min. The lysates collected after cell lysis with RIPA buffer were centrifuged at 14,000 × g at 4°C for 15 min to pellet the agarose and cell debris. The supernatants were then mixed with 2 × Laemmli sample buffer (Bio-Rad) and heated at 95°C for 5 min. Electrophoresis and separation of the protein samples were carried out on 10% sodium dodecyl sulfate polyacrylamide gel, and subsequently transferred onto a nitrocellulose membrane. To block non-specific binding of the antibodies, the nitrocellulose membrane was incubated at room temperature for 1 h with 5% bovine serum albumin (PAA Laboratories, Pasching, Austria) in Tris-buffered saline with 0.1% Tween (TBST). Subsequently, the membrane was incubated with mouse monoclonal antibodies specific for loading control protein β-actin (Santa Cruz, Dallas, Texas), the proliferation marker PCNA (Santa Cruz), and the apoptosis marker poly ADP ribose polymerase 1 (PARP; Cell Signaling, Danvers, Massachusetts) which were all diluted at 1:500 with 5% bovine serum albumin in TBST, overnight at 4°C. The membrane was washed in TBST before and after incubating with the secondary goat anti-mouse IgG-HRP antibody (Santa Cruz) and goat anti-rabbit IgG-HRP antibody (Santa Cruz) for 1h at room temperature. The signal was then developed using the Amersham ECL Prime Western blotting Detection Reagent (GE Healthcare Life Sciences, Uppsala, Sweden) and imaged using the ChemiDocMP imaging system (Bio-Rad).

### Microscopy and image segmentation

3D image stacks of the fixed cells, and the live spheroids and fluorescent beads embedded within the agarose gels, were obtained with the Carl Zeiss LSM 5 LIVE inverted confocal microscope with a ×20 air objective lens (Carl Zeiss Microscopy).

### 3D spheroid expansion stress using digital volume correlation and the Lucas-Kanade method

To derive the displacement field between the far red channel images at two time points, we use the Modified Iterative-Warping Scheme (MIWS) (Besnerais and Champagnat, 2005) extended to three dimensions, a variation of the well-known Lucas-Kanade method (Lucas and Kanade, 1981) for optical flow estimation. This method produces dense displacement fields, with a displacement value for every voxel in the image and has been previously described in another publication (Chew et al., 2018).

For two images *I*_*1*_ and *I*_*2*_ both of *G* ∈ Z^3^ pixels, for each pixel *k* ∈ *G*, we seek a displacement *u(k)* that when applied to an interpolation of *I*_*2*_ minimizes a quadratic error *E*_*k*_ between the pixels *k’* ∈ *N* in a finite neighbourhood *N* ∈ Z^3^ around *k* of each image, weighted by some radial weight function *w*. Using the sum squared difference (SSD) as the error:

$$E_{k}\left(u\left(k\right)\right)=\sum_{k^{\prime }\in N}w\left(k-k^{\prime }\right){\left(I_{1}\left(k^{\prime }\right)-I_{2}\left(k^{\prime }+u\left(k\right)\right)\right)}^{2}$$ (1)

We minimize (1) using a 3D adaptation of the MIWS (Montel et al., 2012), a variant of the Lucas-Kanade method. MIWS takes the first-order Taylor expansion of (1) with respect to *u(k)-u*_*n*_ *(k’)*:

$$E_{k}\left(u\left(k\right)\right)=\sum_{k^{\prime }\in N}w\left(k-k^{\prime }\right){\left(I_{1}\left(k^{\prime }\right)-I_{2}\left(k^{\prime }+u_{n}\left(k\right)\right)-\nabla I_{2}{\left(k^{\prime }+u_{n}\left(k^{\prime }\right)\right)}^{Τ}\left(u\left(k\right)-u_{n}\left(k^{\prime }\right)\right)\right)}^{2}$$ (2)

Taking *∂E*_*k*_ */∂u(k)* = 0, and $I_{2}^{n}$ *(k’) = I*_*2*_ *(k’+ u*_*n*_ *(k’))*, we have:

$H\left(k\right)u\left(k\right)=c\left(k\right)$ , where

$$\begin{array}{l} H\left(k\right)=\sum_{k^{\prime }\in N}w\left(k-k^{\prime }\right)\nabla I_{2}^{n}\left(k^{\prime }\right)\nabla I_{2}^{n}{\left(k^{\prime }\right)}^{Τ}\text{and}\\ c\left(k\right)=\sum_{k^{\prime }\in N}w\left(k-k^{\prime }\right)\nabla I_{2}^{n}\left(k^{\prime }\right)⊗\left(I_{1}\left(k^{\prime }\right)-I_{2}^{n}\left(k^{\prime }\right)+\nabla I_{2}^{n}{\left(k^{\prime }\right)}^{Τ}u_{n}\left(k^{\prime }\right)\right) \end{array}$$ (3)

In this scheme, only one interpolation of *I*_*2*_ needs to be performed per iteration, since the interpolations at each pixel *k* are independent of *k*. The method returns a dense displacement field, i.e., displacement for every voxel.

### Statistical analysis

All data were presented as the mean ± standard deviation (SD) of three independent experiments unless otherwise stated. Student’s t-test was performed to assess the variability between two groups. The means of two datasets are significantly different if the following *p*-values were obtained: *p* < 0.05 (*), *p* < 0.01 (**), n.s = no significant differences.

## Data Availability

The original contributions presented in the study are included in the article/Supplementary Material, further inquiries can be directed to the corresponding authors.
