# Supplementary material for: The role of cell-matrix adhesion and cell migration in breast tumor growth and progression
Source: Front Cell Dev Biol. 2024 Feb 5;12:1339251. doi: 10.3389/fcell.2024.1339251 (PMC10875056; doi:10.3389/fcell.2024.1339251)
Supplement: Supplementary file 1 [file Table1.DOCX]

**The role of cell-matrix adhesion and cell migration in breast tumor growth and progression**

Lor Huai Chong^1,2^*, Ai Kia Yip^1^, Hui Jia Farm^1,3^, Lamees N. Mahmoud^4^, Yukai Zeng^1^, Keng-Hwee Chiam^1^*

^1^ Bioinformatics Institute, A*STAR, 30 Biopolis St, Singapore 138671, Singapore

^2^ School of Pharmacy, Monash University Malaysia, Bandar Sunway, Selangor, 47500, Malaysia

^3^ Department of Computer Science, University of Oxford, Oxford, OX1 3QD, United Kingdom.

^4^ Biomedical Engineering Dept, Faculty of Engineering, Helwan University, Helwan, Cairo, Egypt

*** Correspondence to:** [**chiamkh@bii.a-star.edu.sg**](mailto:chiamkh@bii.a-star.edu.sg) **and** [**chong.lorhuai@monash.edu**](mailto:chong.lorhuai@monash.edu)

**Supplementary Figure**


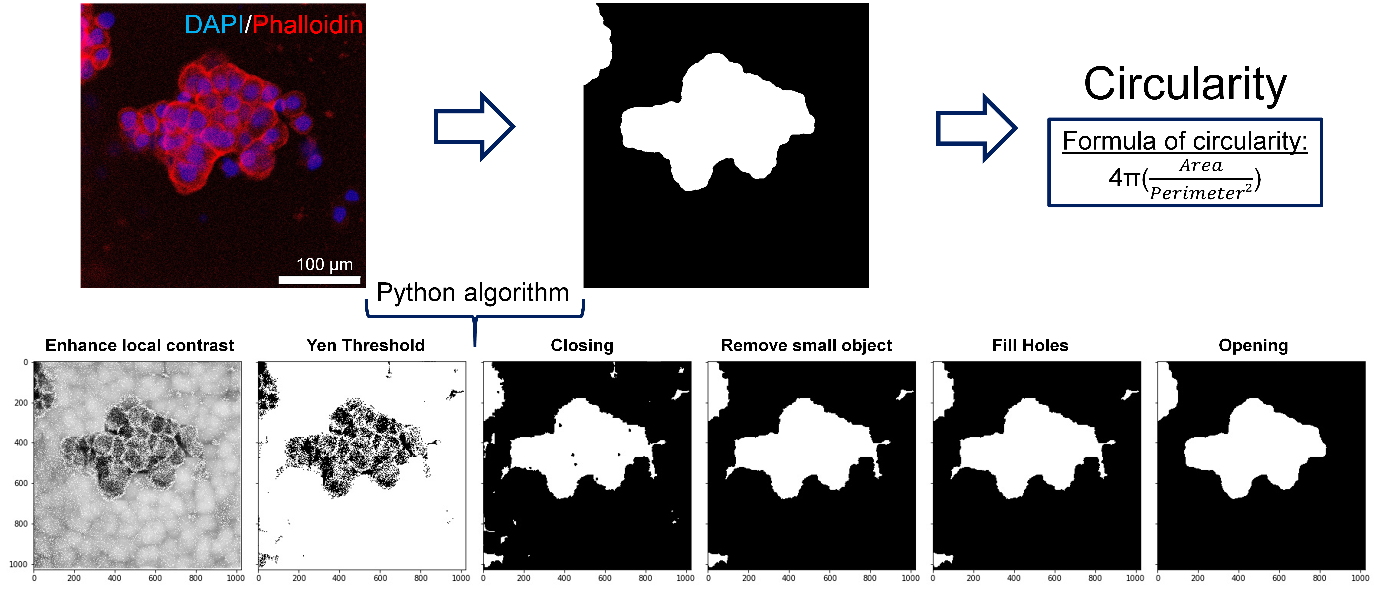


**Figure S1** Illustration of Spheroid Circularity Calculation
